# Family caregivers of elderly with dementia Relationship between religiosity, resilience, quality of life and burden

**DOI:** 10.1590/1980-57642018dn12-040011

**Published:** 2018

**Authors:** Carla Fabiana Carletti Pessotti, Lineu Corrêa Fonseca, Gloria Maria de Almeida Souza Tedrus, Diana Tosello Laloni

**Affiliations:** 1 Psychologist, Master’s Degree in Health Sciences, Pontifícia Universidade Católica de Campinas, Campinas, SP, Brazil; 2 Professor of the Graduate Program in Health Sciences, Pontifícia Universidade Católica de Campinas, Campinas, SP, Brazil; 3 Professor of the School of Psychology, Pontifícia Universidade Católica de Campinas, Campinas, SP, Brazil.

**Keywords:** family caregiver, dementia, quality of life, burden, religiosity, resilience, cuidador familiar, demência, qualidade de vida, sobrecarga, religiosidade, resiliência

## Abstract

There are gaps in knowledge regarding how the family caregiver deals with the burden of caring for elderly people with dementia.

**Objective:**

To evaluate the family caregivers’ perception of quality of life (QoL), burden, resilience and religiosity and relate them with cognitive aspects and occurrence of neuropsychiatric symptoms of elderly with dementia.

**Methods:**

Data from the QoL-AD scale, caregivers’ version, burden interview, resilience scale, Beck depression inventory and PDUREL of 50 family caregivers were correlated with disability assessment for dementia, neuropsychiatric inventory and clinical aspects of 50 elderly with dementia.

**Results:**

Linear regression showed that resilience is related with better perceived QoL (*p*<0.001), severity of dementia (*p*=0.008), higher intrinsic religiosity (IR) (*p*=0.044) and lower occurrence of depressive symptoms (*p*=0.001). Increased burden of family caregivers was associated with a higher occurrence of neuropsychiatric symptoms, education of the elder with dementia, and worse perceived QoL (*p*<0.001). Lower level of organizational religiosity was associated with severity of dementia.

**Conclusion:**

The most resilient caregivers had higher QoL and IR, fewer depressive symptoms, and cared for elders with more severe dementia. Cognitive and sociodemographic aspects, as well as neuropsychiatric symptoms, in the elderly with dementia were associated with QoL and greater caregiver burden.

## RESUMO

Existem lacunas no conhecimento sobre como o cuidador familiar lida com o ônus de cuidar de idosos com demência.

**Objetivo:**

Avaliar a percepção de qualidade de vida (QV), sobrecarga, resiliência e religiosidade dos cuidadores familiares e relacioná-los com aspectos cognitivos e ocorrência de sintomas neuropsiquiátricos em idosos com demência.

**Métodos:**

Dados da escala de QV, versão dos cuidadores, entrevista de sobrecarga, escala de resiliência, inventário de depressão de Beck e PDUREL de 50 cuidadores familiares foram correlacionados com avaliação de incapacidade para demência, inventário neuropsiquiátrico e aspectos clínicos de 50 idosos com demência.

**Resultados:**

A regressão linear mostrou que a resiliência está relacionada a melhor percepção da QV (p<0,001), gravidade da demência (p=0,008), maior religiosidade intrínseca (p=0,044) e menor ocorrência de sintomas depressivos (p=0,001). O aumento da sobrecarga do cuidador familiar esteve relacionado à maior ocorrência de sintomas neuropsiquiátricos, escolaridade dos idosos com demência e pior percepção da QV (p<0,001). Nível inferior de religiosidade organizacional estava relacionado à gravidade da demência.

**Conclusão:**

O cuidador mais resiliente apresenta maior QV e IR, menos sintomas depressivos e cuida de idosos com graus mais severos de demência. Aspectos cognitivos, sociodemográficos e sintomas neuropsiquiátricos em idosos com demência estão relacionados à QV e maior sobrecarga do cuidador.

Dementias are chronic neurodegenerative diseases characterized by progressive cognitive/behavioral decline, cognitive deficits in various domains that are sufficiently severe to compromise occupational or social functioning and independence in activities of daily living.^1^

As the disease progresses, the elderly require help for basic daily activities and, in the most severe stages of the disease, comprehensive care leads to intense biopsychosocial repercussions and burden of caregivers.^2^ In Brazil, family carers predominate in view of the lack of a formal institutional support network.^3^^,^ 4 It is estimated that, in different countries, family support accounts for 90% of care.^5^

It is well known that care can promote negative outcomes for some individuals such as poor health, depressive symptoms, and poor well-being.^6^^-^ 8 Studies suggest that impairment of quality of life (QoL), increased burden and occurrence of depression may be high in dementia caregivers, factors which are frequently related to aspects of the disease such as worsening of cognitive impairment, occurrence of neuropsychiatric disorders as well as impairment of basic activities of daily living.^6^^-^ 9

However, there are still large gaps in knowledge on the use of coping strategies by family caregivers, such as resilience and religiosity as protective factors to reduce burden and promote QoL.

Quality of life according to the World Health Organization is “the individual’s perception of their position in life, in the context of their culture and value systems in which they live, and in relation to their goals, expectations, standards and concerns”.^10^ Resilience has been defined as one’s capacity for successful adaptation when faced with the stress of adversity. Resilience is not invulnerability to stress, but, rather, the ability to recover from negative events.^11^ Perceived burden is a multidimensional and multifaceted concept, and a psychological indicator that designates the caregiver’s attitudes and emotional responses to the demands of caring.^12^

Our hypothesis is that perceived QoL and burden of family caregivers is more related to aspects of religiosity and to more resilient responses and less associated with clinic aspects of the elder with dementia.

Thus, the aim was to evaluate the impact of caring for elderly with dementia regarding perceived QoL, burden and aspects of resilience and religiosity and to relate them to the clinical and cognitive aspects and occurrence of neuropsychiatric and behavioral symptoms of the elders with dementia.

## METHODS

### Participants

The sample consisted of 50 family caregiver-patient dyads. The family caregivers provided informal, unpaid caregiver support to the elderly for more than 6 hours a day and were the sole companions of the elderly for routine medical appointments.

The 50 elderly with dementia over the age of 65 years attended the clinical neurology outpatient clinic of PUC-Campinas Hospital. Dementia was diagnosed by the author (LCF) in accordance with the Diagnostic and Statistical Manual of Mental Disorders^1^ and the National Institute of Neurological and Communicative Disorders and Stroke, and Alzheimer’s Disease^13^ and the recommendations of the National Consensus for the Diagnosis of probable Alzheimer’s Disease.^14^ The Clinical Dementia Rating (CDR)^15^ was applied for staging the severity of dementia.

Only data from the Mini-Mental State Examination (MMSE),^14^ category fluency test (animal VF),^14^ the Clock-Drawing Test^14^ and, CDR^15^ scores were used in this study.

Family caregivers and the elderly with dementia were invited to participate in the study. They were informed about the study and consented to participate by signing the informed consent form. The study was approved by the Human Research Ethics Committee of PUC-Campinas, under protocol nº 1183419.

### Procedures

The family caregivers answered the following instruments:

- Questionnaire for the collection of sociodemographic data: age, education, gender and degree of kinship.
- Resilience Scale (RS):^16^ an instrument that assesses psychosocial adaptation to adversity. It is composed of 25 seven-point Likert items (1=totally disagree; 7=totally agree). The version adapted to the Brazilian Portuguese language and culture was used.^16^
- Quality of life in Alzheimer’s Disease Scale – Caregivers’ Version (QoL-AD):^17^ the instrument consists of 13 items on a four-point scale (1=bad; 4=excellent). The total score ranges from 13 to 52, and higher values indicate higher perceived QoL. The scale was validated and adapted for use in the Brazilian population in 2006.^18^
- Zarit Burden Interview (BI):^19^ this questionnaire consists of 22 questions used to assess burden, health, social and personal life, financial situation, emotional well-being, interpersonal relationships and environment of the caregiver. The questions are answered on a 0-4 scale (0=never; 4=almost always) and the total score ranges from 0 to 88. A higher score indicates greater perceived burden. The version validated for the Brazilian population was used.^20^
- Beck Depression Inventory:^21^ instrument to assess depression. It consists of 21 questions and the total score ranges from 0 to 63. A higher score indicates greater depressive symptoms.
- Duke Religion Index: PDUREL (Portuguese version):^22^^,^ 23 five items rated on a Likert scale that measures three dimensions of religious involvement related to health outcomes: organizational religiosity (OR); non-organizational religiosity (NOR); and intrinsic religiosity (IR). A lower score indicates higher religiosity index. The version validated for the Brazilian population was used.^23^
- Neuropsychiatric Inventory (NPI):^24^ a 10-item questionnaire assessing the frequency and severity of neuropsychiatric symptoms in patients with dementia. Scores for severity of behavior range from 1 to 3 (mild, moderate and severe) and frequency scores range from 1 to 4 (1=occasional; 4=very frequent). The total score (sum of the frequency x intensity of the 10 items) ranges from 0 to 120 points.
- Disability Assessment for Dementia (DAD):^25^ an instrument that quantifies the functional skills in individuals with cognitive deficits for activities of daily living without the help of a caregiver. The total score is obtained by adding up the items and converting them into a percentage. Higher scores indicate less disability in activities of daily living. The version adapted to the Brazilian culture was used.^26^

### Statistical analysis

To describe the profile of the sample according to the variables studied, frequency tables of the categorical variables were built, including absolute and percentage frequency, mean and standard deviation values.

The verification of possible associations between the quantitative variables was estimated using Spearman’s correlation coefficient.

Based on the significant correlations, for multivariate analysis a forward stepwise linear regression analysis was used to assess the results of QoL, burden, resilience and religiosity scores. The best models were selected based on a trade-off between the highest explained variance (R2) and highest cross-validity (adjusted R2).

The data were treated using the software IBM SPSS Statistics, version 22. The significance level adopted for the statistical tests was p<0.05.

## RESULTS

### Sociodemographic, clinical and cognitive aspects

The mean age, formal years of education and gender of family caregivers and elderly with dementia are shown in Table 1. The elderly with dementia were older and had lower levels of education (years of formal schooling) when compared to the family caregivers.

**Table 1:** Sociodemographic and clinical aspects.

		Family caregiver(n=50)	Elder with dementia(n=50)	p-value	Elder with dementia		
					AD (n=34)	Other dementias (n=16)	p-value
Mean age (y, sd)		54.7 (±11.1)	77.1 (±8.1)	0.021*^a^	79.0 (±7.7)	73.2 (±7.5)	0.021*^a^
Mean education (y, sd)		7.76 (±3.9)	3.3 (±2.4)	0.02*^a^	3.5 (±2.8)	2.8 (±1.5)	0.500^a^
Women n (%)		44 (88%)	25 (50%)	0.04*^b^	16 (47%)	9 (56%)	**0.544^b^**
MMSE			10.6 (±8.1)		12.0 (±7.4)	7.6 (±6.1)	0.028*^a^
Clock-drawing test			1.8 (±2.6)		2.2 (±3.0)	1.1 (±1.1)	0.595^a^
Animal VF			4.14 (±3.8)		4.7 (±4.1)	2.8 (±3.0)	0.111^a^
RS		135.6 (±22.5)					
QoL-AD		35.0 (±5.7)					
BI		33.6 (±17.3)					
BDI		12.7 (±11.1)					
PDUREL	IR	3.10 (±1.78)					
	OR	2.46 (±1.45)					
	NOR	4.0 (±1.5)					
NPI			12.2 (±10.2)		9.8 (±8.9)	17.3 (±11.3)	0.022*^a^
DAD			37.4 (±7.9)		36.8 (±95)	38.8 (±1.9)	0.888^a^

AD: Alzheimer disease; MMSE: Mini-mental state examination; animal VF: category fluency test; RS: Resilience Scale; QoL-AD: Quality of life-Alzheimer's Disease scale - caregivers' version; BI: Zarit Burden Interview; BDI: Beck Depression Inventory; PDUREL: Duke University Religion Index-Portuguese version; IR: Intrinsic Religiosity; OR: Organizational Religiosity; NOR: Non-organizational Religiosity; NPI: Neuropsychiatric Inventory; DAD: Disability Assessment for Dementia.

a Mann-Whitney test;

b Chi-square;

* *p*<0.05.

Family caregivers were mainly women who lived with the elderly (86%), with a mean caring time of 4.6 years (± 4.3) and, in most cases, were wives (32%) or daughters (54%).

Fifty elderly with dementia were included; 25 (50%) were female, with a mean age of 77.1 (± 8.1) years. The diagnosis of dementia was as follows: 34 cases (68%) had Alzheimer’s disease and 16 cases had other dementias (vascular dementia in 12 cases, dementia associated with alcoholism and Parkinson’s disease in two cases, each); The severity of dementia was classified in accordance with the CDR as mild in 16 cases, moderate in 18 cases, and severe in 16 cases. The mean score on the MMSE was 10.6 (± 8.1), the mean animal VF score was 4.14 (± 3.8) and the mean score on the clock-drawing test was 1.8 (± 2.6) (Table 1).

### Religiosity, resilience, QoL and burden

The scores on the instruments for the family caregivers (RS, QoL-AD, BI, BDI and PDUREL) and for the elderly with dementia (NPI and DAD) are shown in Table 1.

Significant positive correlation was observed between QoL-AD and RS, suggesting that more resilient family caregivers have better perceived QoL.

Better perceived QoL was correlated with lower scores on the BDI, BI and IR, suggesting that the family caregivers with better QoL had lower perceived burden, a higher IR and fewer depressive symptoms.

There was a significant positive correlation between the BDI and BI, suggesting that family caregivers with lower perceived burden hade fewer depressive symptoms.

Higher resilience was correlated with lower perceived burden, lower occurrence of depressive symptoms and higher IR.

There was no correlation between the age and education of the family caregiver with the QoL-AD, BI, RS, IR and BDI scores.

The significant and non-significant correlations are shown in Table 2.

**Table 2:** Correlation between scores on the QoL-AD, RS, BDI, IR (PDUREL), BI, age and education of the family caregiver.

	QoL-AD		RS		IR		BDI		BI	
	Correlation	*p*	Correlation	*p*	Correlation	*p*	Correlation	*p*	Correlation	*p*
Age	-	0.619	0.191	0.182	-0.116	0.418	0.055	0.702	-0.036	0.803
Education	-0.111	0.442	-0.274	0.053	0.229	0.108	0.042	0.770	0.131	0.363
RS	0.560	<0.000*								
IR	-0.310	0.028*	-0.370	0.008*			0.229	0.108		
BDI	-0.545	<.0001*	-0.364	0.009*	0.009	0.948			0.489	<0.000*
BI	-0.511	<0.000*	-0.366	0.008*	0.160	0.265				

QoL-AD: Quality of life in Alzheimer's Disease scale - caregivers' version; RS: resilience scale; IR: intrinsic religiosity (PDUREL); BDI: Beck depression inventory; BI: burden interview. Spearman's correlation;

* *p*<0.05.

There was a significant negative correlation between the QoL-AD and performance on the clock-drawing test, suggesting that a poorer perceived QoL was associated with worse cognitive performance on the visuospatial ability assessment of the elderly with dementia.

There was a significant correlation between BDI and NPI, suggesting that the presence of depressive symptoms in the family caregivers was associated with the occurrence of behavioral symptoms in the elderly with dementia.

There was no correlation between age, education or the DAD of the demented elderly with the QoL-AD, BI, RS, IR and BDI scores of the family caregiver. The values of the correlations are shown in Table 3.

**Table 3:** Correlation among scores on the QoL-AD, RS, IR (DUREL-P), BDI and BI of the family caregiver and age, education, cognitive, NPI and DAD scores of the elder with dementia.

	QoL-AD		RS		IR		BDI		BI	
	Correlation	*p*	Correlation	*p*	Correlation	*p*	Correlation	*p*	Correlation	*p*
Age	0.138	0.336	0.021	0.881	0.111	0.439	-0.101	0.482	-0.135	0.347
Education	0.090	0.532	0.076	0.599	-0.114	0.427	0.102	0.477	0.219	0.126
MMSE	0.159	0.269	-0.197	0.169	0.072	0.616	0.247	0.082	0.166	0.248
Animal VF	-0.154	0.283	-0.267	0.060	-0.019	0.894	0.229	0.108	0.074	0.608
Clock-drawing test	**-0.280**	0.048*	-0.225	0.114	0.170	0.235	0.221	0.121	0.162	0.259
NPI	0.038	0.791	-0.013	0.925	-0.071	0.622	**0.291**	0.040*	0.200	0.161
DAD	0.136	0.344	0.199	0.164	-0.168	0.242	-0.158	0.270	-0.245	0.085

QoL-AD: Quality of life in Alzheimer's Disease scale - caregivers' version; RS: Resilience Scale; IR: Intrinsic Religiosity (DUREL-P); BDI: Beck Depression Inventory; BI: Zarit Burden Interview; MMSE: Mini-Mental State Examination; Animal VF: category fluency test; NPI: Neuropsychiatric Inventory; DAD: Disability Assessment for Dementia. Spearman's correlation;

* *p*<0.05.

### Multivariate analysis

The linear regression analysis was used to determine the factors that affected resilience, including aspects of the family caregiver (age, formal education, caring time, gender, and BI, BDI, IR and QoL-AD scores) with the age, formal education, and NPI scores, MMSE, clock-drawing test, DAD and CDR of elderly with dementia. The linear regression indicated a significant association between higher resilience and better perceived QoL (*p*<0.001), severe dementia (CDR3) (p=0.008) and higher IR (*p*=0.044). The other aspects were excluded from the equation. The adjusted R2 values and the standardized regression weights are shown in Table 4.

**Table 4:** Multivariate linear regression analysis of higher resilience, better perceived quality of life and burden of the family caregiver.

	RS				QoL-AD				BI		
	Beta (EP)	adjusted R^2^	*p*		Beta (EP)	adjusted R^2^	*p*		Beta (EP)	adjusted R^2^	*p*
QoL-AD	0.47 (0.12)	0.3142	<0.001*						-0.55 (0.11)	0.2618	**<0.001**
CDR (3)	11.15 (3.98)	0.0887	0.008*								
IR (PDUREL)	-0.27 (0.13)	0.0520	0.044*								
RS					0.42 (0.12)	0.3142	<0.001*				
BDI					-0.39 (0.12)	0.1346	0.001*				
Education (elder)									0.31 (0.12)	0.0710	0.011*
NPI									0.29 (0.11)	0.0851	0.013*

RS: resilience scale; QoL-AD: quality of life in Alzheimer's disease scale - caregivers' version; BI: burden interview; CDR: clinical dementia rating; IR: intrinsic religiosity; BDI: Beck depression inventory; NPI: neuropsychiatric inventory.

Higher perceived QoL of the family caregiver was significantly associated with higher resilient response (*p*<0.001) and lower occurrence of depressive symptoms (p=0.001) on the linear regression model analysis (Table 4).

On the regression analysis assessing which factors determined the greatest burden on the family caregiver, the NPI, education of the elder with dementia, and worse perceived QoL (*p*<0.001) were included (Table 4).

On the regression analysis assessing which factors were associated with lower organizational religiosity, only the highest severity of dementia on the CDR remained in the equation (p=0.025). For lower IR, male family caregiver (p=0.011) and higher resilience (p=0.004) remained in the equation. The adjusted R2 values and the standardized regression weights are shown in Table 5.

**Table 5:** Multivariate linear regression analysis of greater organizational religiosity and intrinsic religiosity (PDUREL) of the family caregiver.

	OR				IR		
	Beta (EP)	adjusted R^2^	*p*		Beta (EP)	adjusted R^2^	*p*
CRD (3)	11.19 (4.82)	0.1266	0.025*				
RS					-0.34 (0.11)	0.1375	0.004*
Men (caregiver)					13.43 (5.07)	0.1122	0.011*

OR: organizational religiosity; IR: intrinsic religiosity (PDUREL); CDR: clinical dementia rating; RS: resilience scale.

## DISCUSSION

This study evaluated the family caregivers of elderly with dementia receiving outpatient follow-up care and confirmed the hypothesis that the presence of more resilient responses and religiosity reduces burden and improves QoL. There was also an association between religiosity, resilience and QoL of the caregiver and clinical, cognitive and behavioral aspects of the elder with dementia. However, in contrast with the hypothesis of the study, religiosity and burden of the family caregiver were associated with clinical aspects of the elderly with dementia.

The data from this study confirmed predominance of female family caregivers and of spouses or daughters, corroborating the literature findings showing that care and protection are historically and culturally attributed to women.^4^^,^ 9 ^,^ 27

Our data suggest that better perceived QoL of the family caregiver is associated with a lower occurrence of depressive symptoms, higher IR (internalized belief system as part of life) and more resilient responses, as well as with lower burden. The presence of depressive symptoms in the family caregiver is associated with greater perceived burden and presence of neuropsychiatric and behavioral symptoms in the elderly with dementia. It is known that the family caregiver is exposed to risk factors for depression and anxiety and that the high occurrence of health problems is associated with the chronic stress of caregiving, as well as with the progressive and degenerative nature of dementia.^6^^,^ 8 ^,^ 28 ^,^ 29 Neuropsychiatric disorders are among the main causes of stress, burden and depression of caregivers.^9^ Caregivers of individuals with dementia report more emotional, physical, and financial problems than other caregivers and have an increased risk of mortality.^6^^,^ 29

Similarly to some studies, we found no relationship between the QoL of the family caregivers and age, education, severity of dementia (CDR) or presence of neuropsychiatric symptoms in the elderly with dementia.^9^^,^ 26 ^,^ 28 However, worst cognitive performance (Clock-Drawing Test) was associated with worse perceived QoL by the family caregiver, which suggests that progressive cognitive impairment in dementia can generate demands that renders the task of caring more complex, with consequent deterioration of the caregiver’s QoL. Studies indicate that cognitive impairment is a predictive factor for emotional exhaustion, burden, depression, and impairment of QoL of caregivers.^6^^-^ 9 ^,^ 29 ^,^ 30

There was a relationship between the family caregiver’s better perceived QoL and less perceived burden, suggesting that greater burden compromises caregiver’s QoL, possibly due to daily life restrictions and life context, such as changing routines, leisure activities and social life.^14^^,^ 31 In this study, there was no relationship between QoL and DAD scores. This finding is probably related to factors associated with the family caregivers, such as overestimating functional disability or neglecting the autonomy of the elderly with dementia. It is well known that disability in activities of daily living has a major impact on the perceived QoL of caregivers and the elderly with dementia.^32^

Increased severity of dementia (CDR3) was associated with greater organizational religiosity (involvement of the individual in public religious activities, which can be measured by attendance of church services, religious affiliation, and time spent on prayer and religious meetings) and family caregiver’s resilience. This finding suggests that, despite the significant challenges of the family caregivers in terms of the caring task, studies indicate that having resilient responses is important for preserving the mental health and QoL of these individuals.^7^^,^ 9 ^,^ 29 ^,^ 30 ^,^ 32 Authors suggest that caregivers find comfort and support in religiosity^22^^,^ 32 and that it is an important part of the caring process, acting as a mediator and minimizing the negative impact of the illness. Faith promotes acceptance, serenity, and helps coping with the suffering of the progressive disease.^22^

This study has some limitations. First, our clinic is within a university hospital, but is not a tertiary center. Second, this was a cross-sectional study with a relatively small sample from a single institution. Studies with larger samples are required to assess the impact of the findings of this study. Another limitation of our study was the inclusion of cases with other dementias besides dementia due to Alzheimer.

In summary, the data suggest that more resilient family caregivers have higher QoL and IR, fewer depressive symptoms and care for elderly people with greater severity of dementia. Cognitive, sociodemographic aspects and the presence of neuropsychiatric and behavioral symptoms in the elderly with dementia were associated with QoL and greater burden of the caregiver.
